# Genetic variation associated with cardiovascular risk in autoimmune diseases

**DOI:** 10.1371/journal.pone.0185889

**Published:** 2017-10-05

**Authors:** Pedro P. Perrotti, Adrià Aterido, Antonio Fernández-Nebro, Juan D. Cañete, Carlos Ferrándiz, Jesús Tornero, Javier P. Gisbert, Eugeni Domènech, Benjamín Fernández-Gutiérrez, Fernando Gomollón, Esther García-Planella, Emilia Fernández, Raimon Sanmartí, Jordi Gratacós, Víctor Manuel Martínez-Taboada, Luís Rodríguez-Rodríguez, Núria Palau, Raül Tortosa, Mireia L. Corbeto, María L. Lasanta, Sara Marsal, Antonio Julià, on behalf of the IMID Consortium

**Affiliations:** 1 Rheumatology Research Group, Vall d’Hebron Research Institute, Barcelona, Spain; 2 Department of Medicine, University of Barcelona, Barcelona, Spain; 3 Department of Experimental and Health Sciences, Universitat Pompeu Fabra, Barcelona, Spain; 4 UGC Reumatología, Instituto de Investigación Biomédica (IBIMA), Hospital Regional Universitario de Málaga, Universidad de Málaga, Málaga, Spain; 5 Hospital Clínic de Barcelona and IDIBAPS, Barcelona, Spain; 6 Hospital Universitari Germans Trias i Pujol, Badalona, Spain; 7 Hospital Universitario Guadalajara, Guadalajara, Spain; 8 CIBERehd, Madrid, Spain; 9 Hospital Universitario de la Princesa and IIS-IP, Madrid, Spain; 10 Hospital Clínico San Carlos, IDISSC, Madrid, Spain; 11 Hospital Clínico Universitario, Zaragoza, Spain; 12 Hospital de la Santa Creu i Sant Pau, Barcelona, Spain; 13 Hospital Parc Taulí, Sabadell, Spain; 14 Hospital Universitario Marqués de Valdecilla, Santander, Spain; Peking University First Hospital, CHINA

## Abstract

Autoimmune diseases have a higher prevalence of cardiovascular events compared to the general population. The objective of this study was to investigate the genetic basis of cardiovascular disease (CVD) risk in autoimmunity. We analyzed genome-wide genotyping data from 6,485 patients from six autoimmune diseases that are associated with a high socio-economic impact. First, for each disease, we tested the association of established CVD risk loci. Second, we analyzed the association of autoimmune disease susceptibility loci with CVD. Finally, to identify genetic patterns associated with CVD risk, we applied the cross-phenotype meta-analysis approach (CPMA) on the genome-wide data. A total of 17 established CVD risk loci were significantly associated with CVD in the autoimmune patient cohorts. From these, four loci were found to have significantly different genetic effects across autoimmune diseases. Six autoimmune susceptibility loci were also found to be associated with CVD risk. Genome-wide CPMA analysis identified 10 genetic clusters strongly associated with CVD risk across all autoimmune diseases. Two of these clusters are highly enriched in pathways previously associated with autoimmune disease etiology (TNFα and IFNγ cytokine pathways). The results of this study support the presence of specific genetic variation associated with the increase of CVD risk observed in autoimmunity.

## Introduction

Autoimmune diseases are a group of highly disabling chronic disorders characterized by the activation of multiple immune and inflammatory pathways against the self [1]. Overall, the estimated prevalence of autoimmunity is 5–7% in the general population [2]. Among these, the six autoimmune diseases that are associated with a high socio-economic impact are psoriasis (PS), rheumatoid arthritis (RA), psoriatic arthritis (PA), systemic lupus erythematosus (SLE) and inflammatory bowel diseases (IBDs), including Crohn’s disease (CD) and ulcerative colitis (UC). Importantly, patients with autoimmune diseases have shown to have a higher risk to develop cardiovascular diseases (CVD) compared to the general population [3]. Understanding the genetic and biological mechanisms underlying CVD risk in autoimmunity could therefore be fundamental to develop more efficient preventive and therapeutic strategies.

Autoimmune diseases are genetically complex diseases [4]. In the last years, genome-wide association studies (GWAS) have proven highly successful for identifying a large number of disease susceptibility loci [5]. In IBDs, for example, there are now more than 160 risk loci known to be associated to these autoimmune diseases of the gut [6]. Importantly, GWAS have enabled the identification of a shared genetic risk across autoimmune diseases [7]. However, little is known yet about the genetic variation that contributes to clinically relevant phenotypes within each autoimmune disease.

From a clinical perspective, one of the most important phenotypes in autoimmune diseases is the development of CVD. The marked reduction of the life expectancy observed in autoimmune patients has been clearly associated with an increased risk of CVD [8]. To date, multiple epidemiological and clinical factors have been associated with CVD risk both in autoimmune diseases and in the general population. These include age, dyslipidemia, arterial hypertension and obesity. However, there is increasing evidence that the elevated risk of CVD observed in autoimmune diseases is only partially explained by these classical risk factors [9]. In turn, GWAS in case-control cohorts from the general population have demonstrated the existence of a genetic risk background for CVD [10]. To date, more than 25 GWAS for CVD have been performed, resulting in the identification of more than 100 genetic risk variants [11–13]. So far, however, little is known on the impact of these established genetic variants in the risk of a cardiovascular event in autoimmune diseases.

A large number of the genetic variation associated with the susceptibility to autoimmune diseases involves genes associated with the innate and adaptive immune responses [14]. In particular, these include variation at pathways from pro-inflammatory cytokines like Tumor Necrosis Factor alpha (TNFα) and interferon gamma (IFNγ) [15]. There is increasing evidence that inflammation and immune response activity are also important factors to CVD risk in the general population, both at the genetic and at the functional level [16, 17]. Consequently, genetic variants associated with autoimmune disease susceptibility could also contribute to the increase in CVD risk observed in this group of diseases. To date, however, no comprehensive study of the association between autoimmune susceptibility variants with CVD risk has been performed.

GWAS have revealed that many autoimmune risk loci are associated with multiple autoimmune diseases but not necessarily in the same direction (i.e. a risk allele for a particular autoimmune disease can act as a protective allele for other autoimmune diseases) [7]. These complex cross-phenotype associations underscore the importance of pleiotropy in the genetic epidemiology of autoimmunity. Pleiotropy occurs when a gene or genetic variant affects more than one phenotypic trait. Accordingly, the use of GWAS methods embracing this prevalent property should increase the power to identify relevant genetic variation in autoimmune diseases.

In the present study, we have used a large cohort of patients to investigate the genetics of CVD in six of the most clinically relevant autoimmune diseases. For this objective we have tested, for the first time, the association between established CVD risk loci and the development of CVD in autoimmunity. Conversely, we have also analyzed the association between autoimmune-disease loci and CVD risk. Finally, in order to identify new genetic variation associated with CVD risk across autoimmune diseases, we have performed a genome-wide cross-phenotype analysis. Together, the results of this study provide novel insights into the genetic basis of CVD in autoimmune diseases.

## Materials and methods

### Study population

A total of 6,485 patients were recruited by the Immune-Mediated Inflammatory Disease Consortium (IMIDC) between June 2007 and December 2010 [18]. The IMIDC is a Spanish network of clinical researchers aimed at characterizing the genetic basis of autoimmune diseases. All patients were collected from the outpatient’s clinics of more than 80 departments of rheumatology, dermatology and gastroenterology from 51 Spanish University Hospitals belonging to the IMIDC. The cohort of patients used for the present study included: (i) 1,281 RA patients that fulfilled the American College of Rheumatology (ACR) diagnostic criteria; (ii) 1,123 PS patients that were diagnosed based on the dermatologist clinical criteria; (iii) 989 PA patients that fulfilled the Classification Criteria for Psoriatic Arthritis (CASPAR); (iv) 907 SLE patients that fulfilled four or more of the 1982 revised ACR criteria for SLE classification; and (v) 2,185 IBD patients, including 1,358 and 827 patients that were diagnosed using the Lennard-Jones criteria as having CD and UC, respectively (S1 File). All patients were of Western European descent and with all four grandparents born in Spain. The main features of the different patient cohorts are summarized in S1 Table.

All the procedures were followed in compliance with the principles of the Declaration of Helsinki and informed written consent was obtained from all participants. The study and the consent procedure were reviewed and approved by the Ethics Committee of Clinical Research from Vall d'Hebron Hospital Research Institute.

### Cardiovascular disease phenotype

CVD is a heterogeneous phenotype that includes diverse clinical endpoints. In the present study, CVD patients were defined as having ≥ 1 out of the 3 most frequent cardiovascular phenotypes: coronary heart disease (CHD), cerebrovascular accident (CVA) and peripheral arterial disease (PAD). The medical history of CVD was obtained after a detailed and exhaustive interrogation using standardized medical questionnaires provided. These medical questionnaires were filled by medical specialists from all Spanish Hospitals involved in the IMID Consortium according to the CVD definitions described below.

The CHD phenotype was defined as history of angina, acute myocardial infarction or ischemic heart diseases. The history of angina was assessed as evidence of acute-event symptoms including jaw, arm or chest pain as well as other clinically-related cardiac ischemia symptoms. The history of acute myocardial infarction was examined as the evidence of myocardial necrosis that is consistent with a myocardial ischemia. This was detected by a significant increase/decrease of the levels of the main cardiac biomarkers, together with a characteristic change in the electrocardiogram of the patient. The history of ischemic heart disease was confirmed if the patient fulfilled any of the following conditions: (i) ≥1 major epicardial coronary arteries having more than 70% of obstruction; (ii) history of acute myocardial infarction associated with wall motion abnormality; or (iii) diagnosis of coronary artery disease.

The CVA phenotype was used to characterize those autoimmune disease patients that had a transient ischemic attack or an ischemic/hemorrhagic stroke. The appearance of a transient ischemic attack was defined as a brief neurological dysfunction of the brain (i.e. lasting < 24 hours) caused by a cerebral ischemia without acute infarction. The history of an ischemic/hemorrhagic stroke was defined as an acute episode of focal, cerebral, spinal, or retinal dysfunction caused by infarction/hemorrhage of the central nervous system tissue.

The PAD phenotype was defined as the narrowing of the peripheral arteries that is caused by the presence of atherosclerotic plaques. Claudication symptoms are one of the most important PAD subphenotypes that were used to characterize the CVD phenotype of autoimmune disease patients. Clinical data from surgical and non-surgical interventions like coronary revascularization or percutaneous stenting were not collected in the IMID medical questionnaires and, consequently, these clinical subphenotypes were not evaluated in the present study.

Importantly, to identify genetic variation specifically associated with CVD risk in autoimmunity, all patients that had a cardiovascular event before the date of autoimmune disease diagnosis were excluded from the study (N *=* 127 patients).

### GWAS genotyping and quality control

Genome-wide genotyping data from RA, PS, PA, CD and UC patients were obtained from the five cohorts of autoimmune patients that we previously genotyped [18–22]. For the SLE cohort, whole blood samples (5 mL) were collected from 907 SLE patients. From each sample, genomic DNA was isolated using the Chemagic Magnetic Separation Module I (PerkinElmer, Waltham, MA). Whole genome genotyping in the SLE patient cohort was performed using the same array platform (Illumina Quad610 BeadChip). Genotype calling was performed using the GenomeStudio software (v2011.1, Illumina, San Diego, California, USA).

From the 598,258 SNPs genotyped for each patient, we selected all 582,539 autosomal SNPs for the quality control (QC) analysis. In this analysis, we excluded those SNPs that had a minor allele frequency (MAF) < 0.05 and > 5% of missing data (5.6% SNPs). In order to identify markers deviating from Hardy-Weinberg equilibrium, we used an additional cohort of 1,558 healthy controls from the same population and genotyped with the same array [19]. A total of 1,649 SNPs that deviated significantly from Weinberg equilibrium in this cohort were subsequently removed (0.03% SNPs, *P<*1e-4). To evaluate the presence of potential population stratification in the autoimmune patient cohorts, we used the principal component analysis (PCA) implemented in EIGENSOFT (v4.2) software (v4.2) [23]. Using the first 10 PCs of variation over 10 iterations we identified and discarded 201 patients showing an outlier genetic background (*N*_*RA*_ = 32, *N*_*PS*_ = 50, *N*_*PA*_ = 20, *N*_*SLE*_ = 18, *N*_*CD*_ = 64, *N*_*UC*_ = 17, S1 Fig). After the quality control, a final data set of 506,919 SNPs and 5,317 patients was available for association analysis.

### Selection of candidate markers for CVD risk

#### Genetic variants associated with CVD risk in the general population

Genetic variants previously associated with CVD risk phenotypes (including IHD, CVA or PAD) in the general population (S2 Table) were obtained through a comprehensive electronic data search using PubMed (www.ncbi.nlm.nih.gov/pubmed, 4th February 2016). Only established CVD risk loci (i.e. genetic variants that were significant at the genome-wide scale, *P<*5E-8), were selected for the present study. A total of 115 genome-wide significant variants were identified and subsequently tested for association with CVD in each for the six autoimmune diseases.

#### Genetic variants associated with autoimmune disease susceptibility

Genetic variants associated with disease susceptibility were obtained from recently published GWASs [6, 24–27]. A total of 341 genome-wide significant autoimmune disease variants were identified and tested for association with CVD risk (S3 Table).

### Genotype imputation

The CVD and autoimmune risk SNPs that were not directly genotyped in the GWAS array (*N =* 85 CVD risk SNPs and *N =* 244 autoimmune risk SNPs) were imputed using the data from the six patient cohorts. After pre-phasing the haplotypes of each genomic region using SHAPEIT V2-644 software (Oxford, UK), imputation was carried out using IMPUTE V2 software (Oxford, UK) [28]. Genotype imputation was performed using the dense genotyping data from the Caucasian European cohort (*N =* 379 samples) generated by the 1000 Genomes Project (phase 1, version 3) [29]. Only those SNPs with high quality scores (defined as having an info quality metric > 0.8) were selected for downstream analysis. A total of 73 CVD risk SNPs and 218 autoimmune risk SNPs passed imputation quality control. After the imputation step, a final set of 103 CVD risk SNPs and 315 autoimmune risk SNPs was available for the candidate association analysis.

### Statistical association analysis

Using the final data set of QC-filtered SNPs, the statistical association analysis between each variant and CVD risk was performed using the logistic regression model based on the expected genotype counts and implemented in SNPTEST v2 software (Oxford, UK) [30]. In this method, the allele dosage is defined as follows:

$${Allele\;Dosage}_{i}=\sum_{g=0}^{2}\mathrm{Pr}\left(G=g\right)*g$$

Where *g* represents each genotype of a particular genetic variant *i*, and *Pr(G = i)* is the marginal posterior probability obtained by imputation. The allele dosage therefore can take values between 0 and 2.

Traditional CVD risk factors are known to contribute to CVD risk both in the general population and in patients with autoimmune diseases [3]. Therefore, if inadequately controlled, these clinical and epidemiological risk factors could confound the results from CVD genetic association studies. In order to avoid the effect of these confounding factors, we included the established CVD risk factors as covariates in the statistical association analysis. Accordingly, age, sex, dyslipidemia, arterial hypertension, diabetes mellitus type 2, body mass index, physical inactivity and smoking were included as covariates in the logistic regression model (S1 File). Also, in order to control for genetic ancestry the 2 first PCs of variation were included as covariates in the logistic regression model. The summary statistics of the traditional CVD risk factors for each autoimmune disease are shown in S1 Table.

In order to compare the genetic effect of the associated SNPs across autoimmune diseases, we used the Breslow-Day test for homogeneity of the odds ratio. This method was applied to compare the genetic effect across all autoimmune diseases and also across the two most closely related autoimmune diseases at the clinical level: (i) PS and PA; (ii) CD and UC; and (iii) RA and SLE.

### Genome-wide cross-phenotype analysis of CVD risk

In order to identify genetic patterns associated with CVD risk in autoimmune diseases, we performed a genome-wide cross-phenotype meta-analysis (CPMA) as described previously [7]. Briefly, CPMA is a novel approach that allows the identification of genetic variants associated with multiple phenotypes by integrating statistics from GWAS. First, genome-wide significance P-values for CVD risk were calculated for each autoimmune disease using a total of 507,051 SNPs. The SNPs that were found to be nominally associated with CVD risk were then selected (*P*<0.05, *N =* 10,163 SNPs, here) and used to calculate the CPMA statistic. The CPMA statistic efficiently integrates the evidence from multiple phenotypes and tests for the presence of pleiotropic associations. After selecting the SNPs with significant evidence of pleiotropy (*P*_*CPMA*_<0.01), we filtered those variants that were in moderate to high linkage disequilibrium (r^2^>0.2) in order to avoid the inclusion of redundant signals from neighboring markers.

After identifying the independent SNPs showing higher evidence of pleiotropy, we next analyzed the presence of genetic patterns associated with CVD risk in autoimmune diseases. Following the previously described approach, we computed the SNP-SNP distances and we classified them into four categories of statistical association (i.e. 1>*P*>0.05, 0.05>*P*>0.02, 0.02>*P*>0.005, *P*<0.005) [7]. The categorical matrix defined by these four categories (i.e. each row represents a SNP associated with CVD and each column a particular autoimmune disease) was used to compute the Euclidean distance between each SNP pair using the Gower’s method for discrete data. The resulting matrix of SNP-SNP Euclidean distances was finally used to perform the hierarchical clustering analysis using Ward's method. All the steps from the cross-phenotype analysis were performed using the R statistical software.

For each genetic risk pattern, we computed the statistical significance of the association with CVD risk by combining the association statistics using Fisher's method. According to the number of genetic patterns and autoimmune diseases tested for association with CVD risk, the statistical significance was then corrected for multiple testing using the Bonferroni method [31]. In order to functionally characterize each genetic pattern, we performed a functional enrichment analysis. For this objective, we used the "Hallmark Gene Sets" database (Molecular Signatures Database, Boston, www.broadinstitute.org/gsea/msigdb), a collection of curated sets of genes that show strong coordinated gene expression and represent specific well-defined biological processes. The statistical enrichment analysis for each functional gene set was calculated using the hypergeometric test. The False Discovery Rate (FDR) method was used to account for multiple testing [32].

## Results

### CVD characterization in autoimmune disease cohorts

A total of 140 autoimmune patients with CVD and 5,317 event-free autoimmune patients were identified in the autoimmune disease cohorts of the present study. The epidemiological and clinical characteristics of the present cohort are shown in S1 Table.

### Identification of established CVD risk variants associated also with CVD in autoimmune diseases

We successfully replicated the association between 17 genetic variants and CVD risk in the different autoimmune diseases (*P<*0.05, same risk allele, Table 1). Of relevance, none of the associated SNPs overlapped between the different autoimmune diseases. The most significant association was observed between *HHIPL1* gene (intronic SNP rs2895811) and CVD risk in CD (*P =* 5.62E-4).

**Table 1:** Established CVD risk variants associated with CVD risk in the six autoimmune diseases.

Gene	SNP	Chr	Position	IQM	RA	AID	*P*	OR (95% CI)	AID_BR_	*P*_*BD*_
*BSND*,*PCSK9*	rs11206510	1	55030366	DG	T	UC	0.041	6.56 (1.07–40.09)	CD	0.303
*MIA3*	rs17465637	1	222650187	1.00	C	CD	0.029	6.76 (1.20–37.96)	UC	0.058
*SORT1*,*CELSR2*,*PSRC1*	rs602633	1	109278889	0.96	T	PA	0.027	2.57 (1.11–5.90)	PS	0.108
*OTOL1*,TOMM22P6*	rs6789378	3	162449608	1.00	G	UC	0.004	5.54 (1.72–17.8)	CD	0.014
*OTOL1*,*TOMM22P6*	rs11924705	3	162443828	1.00	T	PA	0.009	2.90 (1.30–6.50)	PS	0.085
*STK32B*	rs7673097	4	5366497	0.99	G	UC	0.039	1.79 (1.01–12.50)	CD	0.935
*MTND1P22*,*GUCY1A3*	rs1842896	4	155590307	DG	T	SLE	0.046	1.96 (1.01–3.80)	RA	0.210
*LPA*	rs10755578	6	160548706	0.99	C	UC	0.027	4.16 (1.17–14.74)	CD	0.632
*ZPR1*	rs964184	11	116778201	DG	G	CD	0.037	1.10 (1.01–4.98)	UC	0.917
*PTCSC3*	rs1952706	14	36205321	0.98	C	SLE	0.036	2.11 (1.05–4.23)	RA	0.053
HHIPL1*	rs2895811	14	99667605	DG	C	CD	5.62E-4	34.11 (4.58–254.06)	UC	0.025
ADAMTS7*	rs3825807	15	78796769	1.00	A	SLE	0.049	1.93 (1.03–3.74)	RA	0.020
*ZFHX3*	rs879324	16	73034779	0.98	A	UC	0.016	4.75 (1.34–16.78)	CD	0.358
UBE2Z*	rs46522	17	48911235	1.00	T	PA	0.012	2.67 (1.24–5.77)	PS	0.027
SMARCA4*	rs1122608	19	11052925	0.98	T	RA	0.039	1.69 (1.03–2.78)	SLE	0.013
*AP3D1*,*SF3A2*,*DOT1L*	rs3803915	19	2160530	DG	C	PA	0.029	3.09 (1.21–126.53)	PS	0.286
*APOE*	rs2075650	19	44892362	DG	G	PA	0.024	3.50 (1.17–10.41)	PS	0.122

Gene: closest mapping gene/s to the associated SNP; SNP: Single Nucleotide Polymorphism; Chr: chromosome; IQM: Information quality metric obtained in the imputation step for the indicated genetic variant (DG indicates that a particular genetic was directly genotyped); RA: allele associated with CVD risk in the original publication; AID: autoimmune disease where the CVD risk variant was replicated; *P*: P-value of association of variant with CVD risk in the autoimmune diseases; OR (95% CI): odds ratio and 95% confidence interval of the association between the SNP and CVD risk; AID_BR_: most closely related autoimmune disease; *P*_*BD*_: significance of Breslow-Day test for heterogeneity of effect of the CVD risk SNP between the two most closely related autoimmune diseases.

* Genes showing a significantly different genetic effect on CVD risk between the two most closely related autoimmune diseases (*P*_*BD*_<0.05).

### Identification of heterogeneous effects on CVD risk across autoimmune diseases

**Fig 1:**
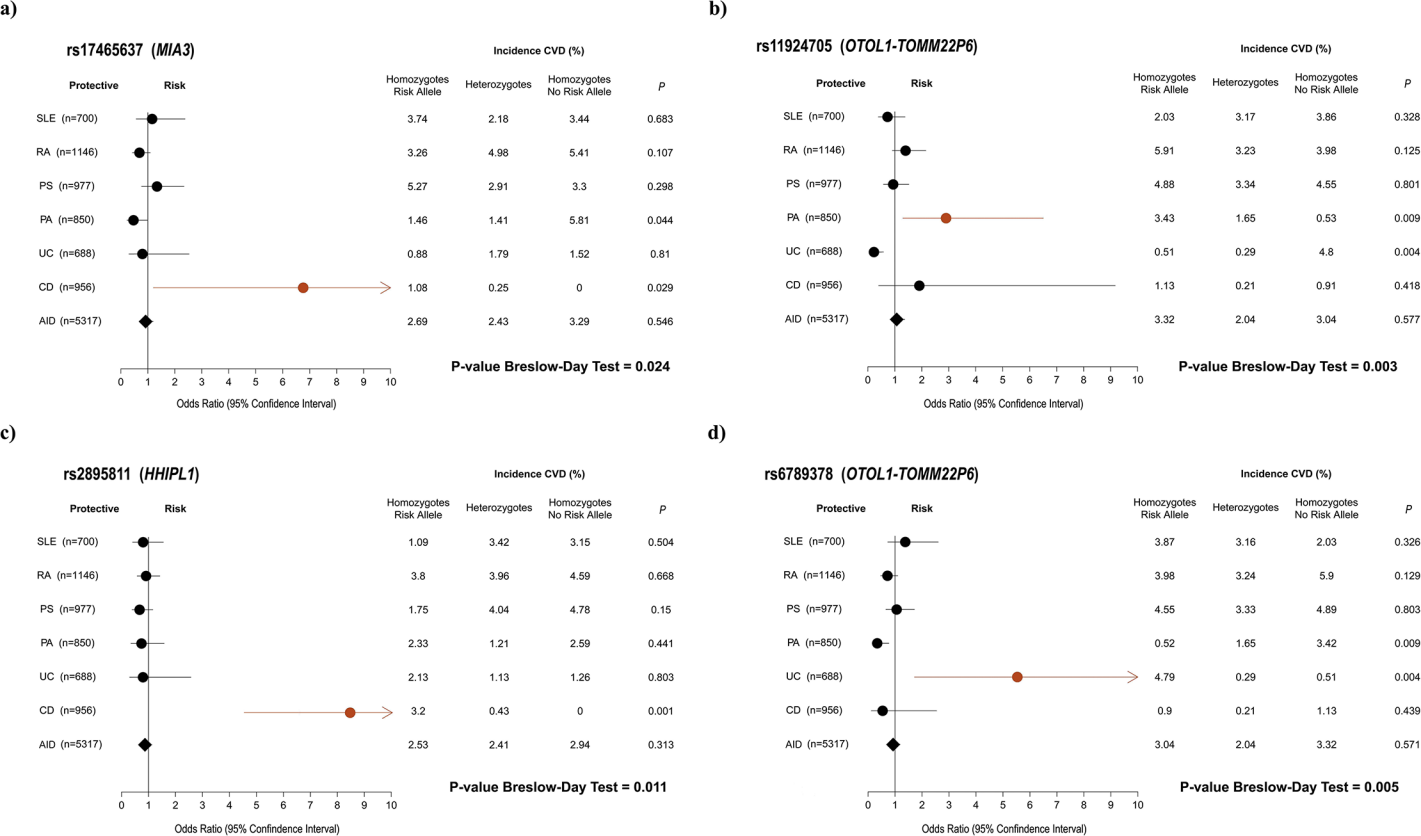
Genetic variants showing heterogeneous genetic effects on CVD risk across autoimmune diseases. For each associated SNP (**A:** rs17465637. **B:** rs11924705. **C:** rs2895811. **D:** rs6789378) the Odds Ratio (OR, black dots) and 95% confidence intervals (horizontal lines) are shown for each of the 6 autoimmune diseases and the combined autoimmune cohort (AID). The SNPs showing a significant association with CVD risk in an autoimmune disease are highlighted in red. For each of the three genotypes of each SNP (risk allele homozygous, heterozygous, non-risk allele homozygous), the incidence of CVD is described in the accompanying table.

From the 17 genetic variants that were significantly associated with CVD risk, we found that 4 SNPs showed significant heterogeneity of genetic effects across the six autoimmune diseases (*P*_*Breslow-Day*_<0.05, Fig 1). Three of these variants displaying significant heterogeneous association with CVD risk were associated with IBDs: SNP rs17465637 (*MIA3* gene; *P =* 0.03; OR = 6.76; 95% CI, 1.21–37.96) and SNP rs2895811 (*HHIPL1* gene; *P =* 0.001; OR = 8.51; 95% CI, 1.80–80.02) were associated with CD, and SNP rs6789378 (*OTOL1-TOMM22P6* locus; *P =* 0.004; OR = 7.52; 95% CI, 2.11–40.78) with UC. Comparing the genetic effect on CVD risk between the most clinically similar autoimmune diseases, we detected significantly different effects in 5 out of the 17 CVD risk SNPs (*P*_*Breslow-Day*_<0.05, Table 1).

### Identification of autoimmune disease risk variants associated with CVD risk

A total of 6 SNPs associated with autoimmune disease risk were also found to be significantly associated with CVD risk (*P<*0.05, Table 2). Four of these variants were IBD risk SNPs and two were markers associated with RA susceptibility.

**Table 2:** Established autoimmune susceptibility variants also associated with CVD risk.

Autoimmune disease	SNP	Chr	Position	IQM	Gene	RA	*P*	OR (95% CI)
Rheumatoid arthritis	rs6732565	2	110850255	1.00	*ACOXL*	A	0.021	1.74 (1.09–2.79)
Rheumatoid arthritis	rs6715284	2	201289674	0.99	*CFLAR-CASP8*	G	0.028	2.35 (1.09–5.04)
Crohn's disease	rs7702331	5	73255307	DG	*LOC105379031*	A	0.029	5.52 (1.19-25-71)
Crohn's disease	rs212388	6	159069404	DG	*TAGAP*	C	0.044	5.51 (1.04–29.11)
Crohn's disease	rs3897478	1	119908567	1.00	*ADAM30*	T	0.038	1.83 (1.01–78.24)
Ulcerative colitis	rs4728142	7	128933913	DG	*IRF5*	A	0.011	4.68 (1.43–15.30)

SNP: Single Nucleotide Polymorphism; Chr: chromosome; IQM: Information quality metric obtained in the imputation step for the indicated genetic variant (DG indicates that a particular genetic was directly genotyped); Gene: closest mapping gene/s to susceptibility variant; RA: allele associated with autoimmune disease risk; *P*: significance of association between the indicated genetic variant and CVD risk; OR (95%CI): Odds ratio and 95% confidence interval for the observed association.

### Genetic patterns associated with CVD risk in autoimmune diseases

A total of 765 SNPs were found to have a significant multi-phenotype association (*P*_*CPMA*_<0.01, *P*_*Binomia*l_<5.74E-28). After removing the markers in linkage disequilibrium (r^2^>0.2), a final set of 406 independent pleiotropic SNPs was used for downstream analysis (S4 Table). The global sharing of genetic associations with CVD risk across the six autoimmune diseases is represented in S2 Fig.

**Fig 2:**
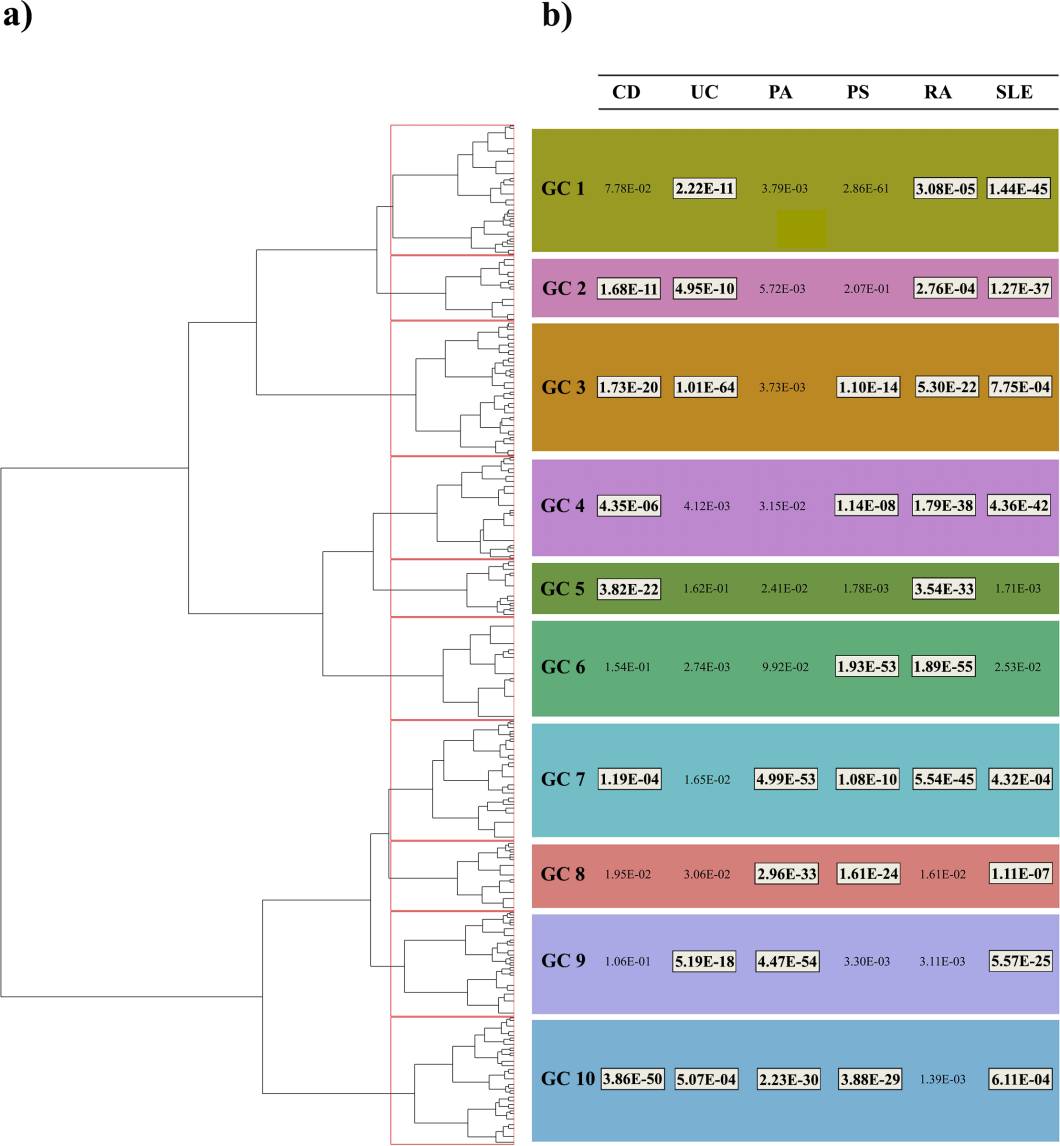
Identification of the genetic clusters associated with CVD risk across autoimmune diseases. **A:** Hierarchical clustering dendrogram showing the similarity between SNPs with significant evidence of pleiotropy with CVD risk. **B:** Statistical significance of the association between each genetic cluster and CVD risk for the six autoimmune diseases. Significant associations after multiple test correction are highlighted in white boxes. Abbreviations: GC: genetic cluster.

Using the clustering approach on the 406 pleiotropic variants, we identified 10 different genetic clusters (GC) associated with CVD risk in autoimmune diseases (Fig 2A and S5 Table). For each autoimmune disease, the statistical association between each GC and CVD risk is shown in Fig 2B. From the 10 GCs identified, we found that 3 GCs were significantly enriched in 7 sets of functionally-related genes (Table 3 and S6 Table). Importantly, after multiple testing correction, GC4 and GC7 were found to be significantly enriched in genes that are involved in immune-related pathways strongly associated with the autoimmunity etiopathogenesis like the TNFα (*P*_*FDR*_ = 0.02) and INFγ cytokine pathways (*P*_*FDR*_ = 0.02), respectively.

**Table 3:** Functional characterization of the genetic clusters associated with CVD risk across autoimmune diseases.

GC	Gene Set	Description	Gene#	*P*_*HYPER*_	*P*_*FDR*_
GC 4	Apical surface	Genes encoding proteins over-represented on the apical surface of epithelial cells.	44	7,17E-4	0.019
GC 4	TNFα signaling via NFKB	Genes regulated by NFKB in response to TNF.	200	7,66E-4	0.019
GC 6	Estrogen response early	Genes defining early response to estrogen.	200	5,21E-4	0.013
GC 6	Estrogen response late	Genes defining late response to estrogen.	200	5,21E-4	0.013
GC 7	Adipogenesis	Genes up-regulated during adipocyte differentiation.	200	1,01E-3	0.017
GC 7	Complement	Genes encoding components of the complement system.	200	1,01E-3	0.017
GC 7	Interferon Gamma response	Genes up-regulated in response to IFNγ.	200	1,01E-3	0.017

GC: genetic cluster; Gene Set: Hallmark gene set associated with CVD risk; Gene#: number of genes in the indicated gene set; *P*_*HYPER*_: significance of association between the indicated GC and biological process using the hypergeometric test; *P*_*FDR*_: significance of the functional association after correcting for multiple testing using the False Discovery Rate method.

## Discussion

CVD represents one of the most clinically relevant phenotypes in autoimmune diseases. In this work, we have used a large multicenter cohort of six autoimmune diseases to characterize the genetic risk for CVD in this group of diseases. First, we have replicated the association of established CVD risk loci with CVD in autoimmune diseases. Second, we have found that multiple autoimmune susceptibility loci are also associated with CVD risk. Finally, using a cross-phenotype genome-wide analysis we have identified genetic patterns significantly associated with CVD risk in autoimmune diseases. Taken together, these findings support the existence of a differential genetic component for CVD risk in autoimmunity.

We have successfully validated the association of 17 established CVD risk SNPs in autoimmune diseases. This result confirms that genetic variation associated with the development of cardiovascular events in the general population can also be associated with CVD risk in this group of chronic inflammatory diseases. Several of these associated genes have relevant functional implications that could link both disease etiopathogenesis. For example, *ADAMTS7* (rs3825807) encodes a metalloproteinase that has been associated with arthritis and also with the thickening of the neointima occurring in cardiovascular events [33, 34]. The association between CVD risk and *SMARCA4* (rs1122608) in RA patients is also consistent with the disease pathogenesis. RA is characterized by the predominant infiltration of CD4+ T lymphocytes in the synovial joints. SMARCA4 (also known as BRG1) has been shown to be essential for the differentiation of Th1 CD4+ T cells [35], which is the predominant T cell subset in RA synovial joints. Conversely, variation at *SMARCA4* gene has been recently associated to low-density lipoprotein cholesterol and apolipoprotein B levels [36], thereby providing a direct etiological contribution to CVD risk.

One of the most important findings of the present study is the identification of differential effects in established CVD risk genes across autoimmune diseases. This result suggests that the proinflammatory state that characterizes each autoimmune disease modulates specifically the CVD risk conferred by the known CVD genetic risk factors. For example, the genetic risk variant rs2895811 at *HHIPL1* gene showed a significantly stronger association with CVD risk in CD than in the other five autoimmune diseases. The protein encoded by this gene participates in the Hedgehog (HH) signaling pathway, a biological process known to be involved in the pathogenesis of IBDs [37]. Very recently, this signaling pathway has been shown to play also a cardio-protective role in cardiomyocytes [38]. This result therefore suggests that the pathologic role of the HH signaling pathway in CVD is enhanced by the specific autoimmune processes activated in CD and, therefore, that CD patients carrying the risk allele for the *HHIPL* locus have a higher probability to develop a cardiovascular event than patients with the non-risk allele. To our knowledge, it is the first time that genetic risk factors for CVD have been shown to be modulated by the presence of an autoimmune disease.

The use of GWAS to identify new genetic risk loci for autoimmune diseases has greatly improved our understanding of the genetic basis of autoimmunity risk [39]. Together with the characterization of the missing heritability, the challenge ahead is to investigate whether these autoimmune disease risk loci are also associated to the most clinically relevant disease phenotypes. CVD is clearly one of the most relevant phenotypes since it has a direct impact in the patient's life expectancy. In the present study we have analyzed the association between known susceptibility variants for autoimmune diseases and CVD risk. For most of these loci it is the first time that this association has been tested. Despite immune activation is now considered a necessary biological process for CVD development [40], to date, very few established CVD risk genes are known to be associated with immunity. Our findings support the existence of a genetic risk component for CVD that is functionally associated with the immune response.

In the association analysis between candidate loci and CVD risk, we were able to identify a significant association for 6 out of 315 autoimmune-associated SNPs and 17 out of 103 CVD-associated SNPs. Although GWAS are a very powerful approach to identify new disease risk genes, this methodology is not always able to identify the exact causal variant [41]. The genetic variation analyzed in the present study (i.e. reference SNPs for both CVD and autoimmunity risk) could be in linkage disequilibrium with the exact causal SNP rather than being the precise causal variant itself. Also, different genetic variation at the same gene has been associated with multiple autoimmune diseases [42]. This suggests that causative SNPs for CVD could differ between diseases, which is consistent with the differences observed across the six autoimmune diseases analyzed. Future studies using independent cohorts of CVD-characterized autoimmune disease patients will provide deeper insights into the disease-specific genetic basis for CVD risk in autoimmunity.

In the present study we have also identified the existence of genetic patterns associated with CVD risk across autoimmune diseases. Based on the existence of pleiotropic effects among genetic variation associated with autoimmune disease risk, we have been able to combine the results across six autoimmune diseases and identify 10 genetic patterns associated with CVD risk. Of relevance, two of these genetic patterns are significantly enriched in genes from immune response pathways that are crucial for this group of autoimmune diseases: TNFα and IFNγ cytokine pathways. The GC enriched in TNFα pathway genes was found to be very strongly associated with CVD risk in RA and SLE (*P* = 1.8E-38 and *P* = 4.36E-42, respectively). TNFα is one of the main mediators of multiple cardiovascular pathological mechanisms underlying CVD [15]. In addition, TNFα is one of the most abundant cytokines produced by the inflamed synovium in RA. Blocking TNFα systemically has proven to be a highly efficacious approach to treat RA, and is currently the predominant biologic therapy after failure of conventional disease-modifying anti-rheumatic drugs. Consequently, genetic variation influencing this shared pathway could contribute to increase the risk of a cardiovascular event in RA patients. Evidence from national registers suggesting that patients treated with anti-TNF agents have a lower probability to develop CVD supports this possibility [43]. In order to identify the therapeutic potential of these results, further studies evaluating the role of TNFα in the development of CVD in RA and SLE patients as well as the role of IFNγ in PA and RA patients are needed.

The functional enrichment analysis of the genetic clusters associated with CVD risk in autoimmune diseases also showed a genetic cluster (GC6) significantly enriched in genes that participate in the estrogen response. For decades, it has been known that there is a higher prevalence of CVD in males compared to females. This observation has contributed to the hypothesis that sex-related hormones like estrogens, which are also potent stimulators of autoimmunity, contribute to the risk of developing CVD [44, 45]. The finding that estrogen response genes (GC6) are associated with CVD in PS and RA suggests that, within these two autoimmune diseases, the role of sex hormones in increasing CVD risk is partially mediated by genetic variation at genes participating in the estrogen response.

This study represents the first comprehensive analysis of genetic variation associated with CVD risk in autoimmune diseases. Our findings provide new insights into the molecular mechanisms that contribute to increase the risk of a cardiovascular event in autoimmune diseases. Nevertheless, this study has limitations. Despite that the sample size used in each of the six autoimmune diseases is large, the number of patients with CVD events is relatively low. The main reason is the low prevalence of this severe phenotype within each disease cohort but also the exclusion of patients that had a CVD event before the autoimmune disease initiation. This former selection criteria, although reduced the total number of CVD patients available for analysis, was essential to identify the genetic variation that is relevant for CVD in autoimmunity. Another limiting factor in this study is the heterogeneity in the CVD phenotype. Similar to previous studies, we included patients with different forms of CVD, and this could have prevented the identification of additional genetic associations. It is likely that stratified analyses for each of the different CVD subphenotypes will help to reveal new risk variation, although this would require much larger patient cohorts than the ones used in the present study. Future studies integrating data from different multicenter cohorts like the present one will be necessary.

In the present study we have performed a comprehensive analysis of genetic variation associated with CVD risk in autoimmune diseases. Using large cohorts of six autoimmune diseases that are associated with a high socio-economic impact, we have replicated the association of established CVD risk loci. Importantly, we have found that several of these loci are differentially associated across autoimmune diseases. This result supports the hypothesis that the disease-specific inflammatory component contributes differentially to increase CVD risk in autoimmunity. In addition, we have identified genetic variation previously associated with autoimmune diseases susceptibility that is also associated with CVD risk. This result could contribute to explain the increased prevalence of CVD observed in autoimmune diseases. Replication in additional independent cohorts will be needed to confirm this finding. Finally, we have identified the major genetic patterns associated with CVD risk in autoimmune diseases. These results suggest novel biological mechanisms underlying the development of CVD in autoimmunity.

## Supporting information

S1 File The supplementary information is available in the “*Supporting Information”* file. (PDF)

S1 Fig Principal component analysis of the autoimmune disease cohorts. (PDF)

S2 Fig Global sharing of genetic variants associated with CVD risk across autoimmune diseases. (PDF)

S1 Table Main epidemiological and clinical features of the autoimmune cohorts included in the present study. (ODS)

S2 Table Reference publications of GWAS on CVD risk. (ODS)

S3 Table Genetic variants associated with autoimmune disease risk. (ODS)

S4 Table Independent genetic loci nominally associated with CVD risk in multiple autoimmune diseases. (ODS)

S5 Table Genetic content of the 10 genetic clusters associated with CVD risk across autoimmune diseases. (ODS)

S6 Table Functional characterization of the 10 genetic clusters associated with CVD risk across autoimmune diseases. (ODS)
