# Supplementary material for: Genetic variation associated with cardiovascular risk in autoimmune diseases
Source: PLoS One. 2017 Oct 5;12(10):e0185889. doi: 10.1371/journal.pone.0185889 (PMC5628882; doi:10.1371/journal.pone.0185889)
Supplement: S1 File — (PDF) [file pone.0185889.s001.pdf]

SUPPLEMENTARY MATERIALS AND METHODS

INDEX

1.- Study population 2

2.- CVD phenotypes 3

3.- References 4

1.- Study population

A total of 6,485 patients were recruited from the Immune-Mediated Inflammatory Disease Consortium (IMIDC).[1] The IMIDC is a Spanish network of clinical researchers aimed at characterizing the genetic basis of autoimmune diseases. All patients were collected from the outpatient's clinics of more than 80 departments of rheumatology, dermatology and gastroenterology from 51 Spanish University Hospitals belonging to the IMIDC. The patients included in the autoimmunity cohort used for the present study are described as follows:

- Rheumatoid arthritis (RA, *n* = 1,281patients): All RA patients satisfied the American College of Rheumatology (ACR) classification criteria for RA[2] and had >2 years of follow-up since diagnosis.
- Psoriasis (PS, *n* = 1,123 patients): All PS patients had >18 years at the time of sample collection and were diagnosed based on dermatologist clinical criteria,[3] with chronic plaque psoriasis affecting torso and/or extremities with at least one year of duration.
- Psoriatic arthritis (PA, *n* = 989 patients): All PA patients fulfilled the Classification Criteria for Psoriatic Arthritis (CASPAR)[4] and were >18 years old, although the disease could have started earlier in life.
- Systemic lupus erythematosus (SLE, *n* = 907 patients): All SLE patients were diagnosed using the 1982 revised ACR criteria for SLE diagnosis,[5] were >16 years old at the time of sample collection and had >3 years of evolution from the diagnosis date.

- Inflammatory bowel disease (IBD, n = 2,185 patients): IBD patients include 1,358 CD patients and 827 UC patients, which were >18 years old and diagnosed using the Lennard-Jones criteria.[6]

2.- CVD Phenotypes

Clinical and epidemiological data were collected following a standard protocol of medical questionnaires. Clinical data about traditional CVD risk factors was ere exhaustively collected using the medical questionnaires. Using these data, the traditional CVD risk factors were defined for autoimmune disease patients as follows:

- Arterial hypertension. Was diagnosed based on usage of ongoing therapy for hypertension, systolic blood pressure ≥ 140 mm Hg and/or diastolic blood pressure ≥ 90 mm Hg.[7]
- Diabetes mellitus type 2. Fasting plasma glucose >126 mg/L (7.0 mol/L) or 2-hour plasma glucose >200 mg/dL (11.1 mmol/L) during an oral glucose tolerance test.[8]
- Dyslipidemia. Presence of one or more of the following conditions: triglyceride (TG) > 200 mg/dL, high-density lipoprotein (HDL-C) cholesterol < 40 mg/dL, low-density lipoprotein (LDL) cholesterol > 130 mg/dL and treatment with antilipemic agents.[9]
- Body mass index (BMI). Body mass (Kg) divided by the square of the body height (m).[10]
- Smoking. Smokers at the moment of sample collection.
- Physical inactivity. Full-time sitting-down/standing jobs (with reduced physical activity) and free-time with no/occasionally physical exercise.

3.- References

1. Julia A, Domenech E, Chaparro M, et al. A genome-wide association study identifies a novel locus at 6q22.1 associated with ulcerative colitis. *Human molecular genetics.* 2014;23:6927-6934.
2. Arnett FC, Edworthy SM, Bloch DA, et al. The American Rheumatism Association 1987 revised criteria for the classification of rheumatoid arthritis. *Arthritis and rheumatism.* 1988;31:315-324.
3. Boehncke WH, Schon MP. Psoriasis. *Lancet (London, England).* 2015;386:983-994.
4. Taylor W, Gladman D, Helliwell P, Marchesoni A, Mease P, Mielants H. Classification criteria for psoriatic arthritis: development of new criteria from a large international study. *Arthritis and rheumatism.* 2006;54:2665-2673.
5. Tan EM, Cohen AS, Fries JF, et al. The 1982 revised criteria for the classification of systemic lupus erythematosus. *Arthritis and rheumatism.* 1982;25:1271-1277.
6. Lennard-Jones JE. Classification of inflammatory bowel disease. *Scandinavian journal of gastroenterology. Supplement.* 1989;170:2-6; discussion 16-19.
7. Jones DW, Hall JE. Seventh report of the Joint National Committee on Prevention, Detection, Evaluation, and Treatment of High Blood Pressure and evidence from new hypertension trials. *Hypertension.* 2004;43:1-3.
8. Ryden L, Grant PJ, Anker SD, et al. ESC Guidelines on diabetes, pre-diabetes, and cardiovascular diseases developed in collaboration with the EASD: the Task Force on diabetes, pre-diabetes, and cardiovascular diseases of the European Society of Cardiology (ESC) and developed in collaboration with the European Association for the Study of Diabetes (EASD). *European heart journal.* 2013;34:3035-3087.
9. Executive Summary of The Third Report of The National Cholesterol Education Program (NCEP) Expert Panel on Detection, Evaluation, And Treatment of High Blood Cholesterol In Adults (Adult Treatment Panel III). *Jama.* 2001;285:2486-2497.
10. Dong J, Chen Y, Tang Y, et al. Body Mass Index Is Associated with Inflammatory Bowel Disease: A Systematic Review and Meta-Analysis. *PloS one.* 2015;10:1-19.
