# Supplementary material for: Genetic variation associated with cardiovascular risk in autoimmune diseases
Source: PLoS One. 2017 Oct 5;12(10):e0185889. doi: 10.1371/journal.pone.0185889 (PMC5628882; doi:10.1371/journal.pone.0185889)
Supplement: S1 Fig — (PDF) [file pone.0185889.s002.pdf]

Figure S1. Principal component analysis of the autoimmune disease cohorts.

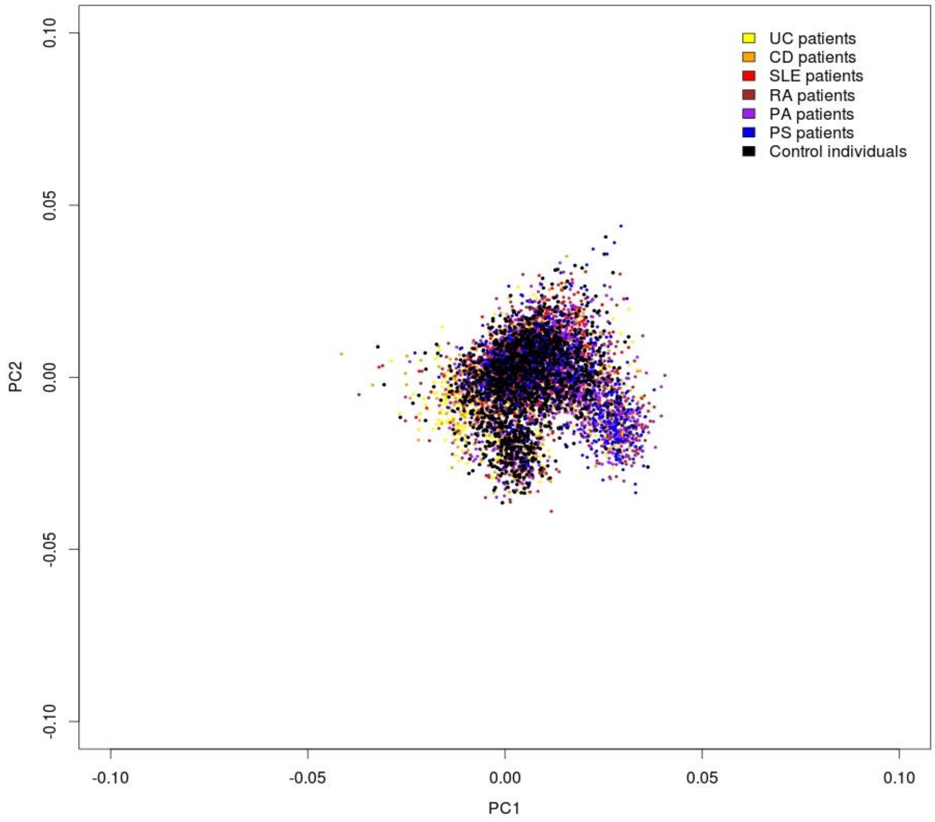

The autoimmune disease patients and healthy controls are plotted according to their first (PC1) and second (PC2) principal components that were estimated using the EIGENSTRAT software. Patients with an outlier genetic variation (i.e. >6 standard deviations in any of the 10 PCs of variation, N=201) were identified and excluded from downstream analysis.
