# Supplementary material for: Genetic variation associated with cardiovascular risk in autoimmune diseases
Source: PLoS One. 2017 Oct 5;12(10):e0185889. doi: 10.1371/journal.pone.0185889 (PMC5628882; doi:10.1371/journal.pone.0185889)
Supplement: S2 Fig — (PDF) [file pone.0185889.s003.pdf]

Figure S2. Global sharing of genetic variants associated with CVD risk across autoimmune diseases.

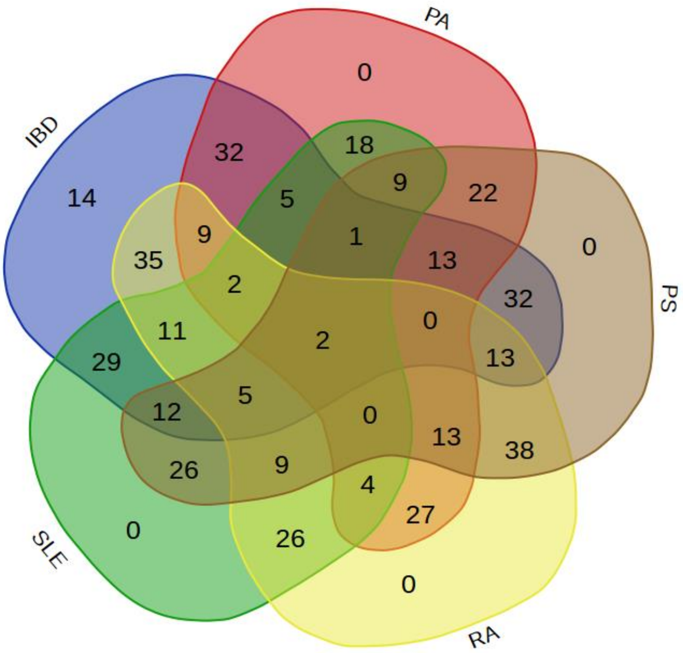

Venn diagram representing the genetic overlap of the SNPs associated with CVD risk across the six prevalent autoimmune diseases considered in this study. IBD: inflammatory bowel diseases; PA: psoriatic arthritis; PS: psoriasis; RA: rheumatoid arthritis; SLE: systemic lupus erythematosus.
